# The Serine/Threonine Protein Phosphatase 2A (PP2A) Regulates Syk Activity in Human Platelets

**DOI:** 10.3390/ijms21238939

**Published:** 2020-11-25

**Authors:** Stephanie Makhoul, Elena Kumm, Pengyu Zhang, Ulrich Walter, Kerstin Jurk

**Affiliations:** 1 Center for Thrombosis and Hemostasis (CTH), University Medical Center Mainz of the Johannes Gutenberg University Mainz, D-55131 Mainz, Germany; stephaniemakhoul@live.com (S.M.); elena.kumm@uni-mainz.de (E.K.); pengyu.zhang@isas.de (P.Z.); 2 Leibniz-Institut für Analytische Wissenschaften, D-44227 Dortmund, Germany

**Keywords:** protein phosphatase 2A, spleen tyrosine kinase (Syk), platelets, glycoprotein VI, glycoprotein Ibα

## Abstract

Distinct membrane receptors activate platelets by Src-family-kinase (SFK)-, immunoreceptor-tyrosine-based-activation-motif (ITAM)-dependent stimulation of spleen tyrosine kinase (Syk). Recently, we reported that platelet activation via glycoprotein (GP) VI or GPIbα stimulated the well-established Syk tyrosine (Y)-phosphorylation, but also stoichiometric, transient protein kinase C (PKC)-mediated Syk serine(S)297 phosphorylation in the regulatory interdomain-B, suggesting possible feedback inhibition. The transient nature of Syk S297 phosphorylation indicated the presence of an unknown Syk pS297 protein phosphatase. In this study, we hypothesize that the S-protein phosphatase 2A (PP2A) is responsible for Syk pS297 dephosphorylation, thereby affecting Syk Y-phosphorylation and activity in human washed platelets. Using immunoblotting, we show that specific inhibition of PP2A by okadaic acid (OA) alone leads to stoichiometric Syk S297 phosphorylation, as analyzed by Zn^2+^-Phos-tag gels, without affecting Syk Y-phosphorylation. Pharmacological inhibition of Syk by PRT060318 or PKC by GF109203X only minimally reduced OA-induced Syk S297 phosphorylation. PP2A inhibition by OA preceding GPVI-mediated platelet activation induced by convulxin extended Syk S297 phosphorylation but inhibited Syk Y-phosphorylation. Our data demonstrate a novel biochemical and functional link between the S-protein phosphatase PP2A and the Y-protein kinase Syk in human platelets, and suggest that PP2A represents a potential enhancer of GPVI-mediated Syk activity caused by Syk pS297 dephosphorylation.

## 1. Introduction

Human platelets are small anucleate blood cells, which are essential components of the hemostasis system and widely recognized as circulating sentinels of the vessel wall [1,2,3]. They prevent blood loss during vascular injury and have important roles in immunity and wound healing, but harmful effects in cancer/metastasis, thrombotic, inflammatory, and immune pathologies [3,4]. Physical, metabolic, inflammatory, and infectious vascular injuries result in newly exposed or altered vessel wall proteins, such as collagen, von Willebrand factor (vWF), fibrin, and podoplanin, which recruit platelets via distinct adhesion receptors/membrane glycoproteins (GP), such as GPVI, GPIb-V-IX, C-type lectin-2 (CLEC-2), and integrin α_IIb_β_3_. This initial adhesion leads to platelet activation with multiple responses, including cytoskeletal remodeling, integrin α_IIb_β_3,_ activation, degranulation, synthesis/release of thromboxane A2 (TxA2), and surface exposure of anionic phospholipids. Functionally, this results in platelet shape change, enhanced adhesion, aggregation, and coagulant activity to form a thrombus [5,6]. These properties are tightly controlled by numerous extracellular hormones, vasoactive factors, and adhesive proteins, which activate, modulate, or inhibit these vital platelet functions. Whereas the adhesion molecules vWF, collagen, fibrin, and podoplanin bind to and activate platelet membrane-spanning adhesion receptors and subsequently stimulate Src-family tyrosine protein kinases (SFKs) [4,6,7], a second class of soluble platelet agonists stimulate specific G-protein-coupled receptors (GPCRs) [8,9]. GPCR-coupled agonists, such as ADP, thrombin, and TxA2 stimulate phospholipase Cß (PLCβ), elevate cytosolic Ca^2+^ concentration, and activate Ca^2+^-dependent protein kinases and protein kinase C (PKC), thereby inducing platelet activation [8,9]. Endothelial-derived inhibitory factors, such as prostacyclin (PGI_2_) and nitric oxide (NO), decrease many of these platelet-activating pathways via the elevation of cyclic adenosine monophosphate (cAMP) and cyclic guanosine monophosphate (cGMP), respectively, and stimulation of their target protein kinases [10,11]. While the extracellular network of factors controlling platelet function(s) is well appreciated, leading to the successful clinical development of antiplatelet drugs [12,13], a similar understanding of the intracellular networks and their interactions is lagging behind. However, increasing evidence is documenting that the intracellular effects of extracellular factors are mediated by a tightly controlled system of interacting signaling molecules, including regular and small G-proteins, second messengers, protein kinases, protein phosphatases, and their substrates [14,15,16]. Global proteomic analyses of both human and murine platelets established that these anucleate cells have, similar to most other cells, a repertoire of hundreds of protein kinases/phosphatases [16,17,18].

Several members of the SFKs, such as Src, Lyn, Yes, Fyn, and feline Gardner-Rasheed sarcoma viral oncogene homolog (FGR), are highly expressed in human platelets and are important components of adhesion-dependent platelet activation mediated by GPVI, GPIb-V-IX, CLEC-2, and integrin α_IIb_β_3_ [4,6,7]. A crucial effector system of SFKs in platelets, but also in immune cells, is the soluble spleen tyrosine kinase (Syk), which contains two N-terminal src homology 2 (SH2) domains and a C-terminal kinase domain separated by an interdomain-B [19,20,21]. Upon stimulation of immune cells and platelets, SFKs are activated and phosphorylate other proteins/protein kinases (also Syk) at tyrosine residues, including membrane proteins with the immunoreceptor tyrosine-based activation motif (ITAM). ITAM proteins are actually phosphorylated at two neighboring Y-phosphosites, which then efficiently recruit the 2-SH2-domain-containing Syk from the cytosol to the cell membrane. The entire process activates Syk via two distinct, overlapping mechanisms, the ITAM-dependent Syk recruitment to the membrane and phosphorylation of critical Syk phosphosites [21,22,23,24]. In human platelets, ITAM-dependent Syk activation is mediated by the Fc receptor γ-chain and the low-affinity IgG receptor FcγRIIa [25,26]. An important Syk substrate is the phospholipase Cγ2 (PLCγ2), which is activated by Syk-catalyzed tyrosine phosphorylation, leading to the generation of multiple second messengers and platelet responses, including integrin activation, secretion, and TxA2 release [6,21]. The intrinsic Syk Y-phosphosites closely associated with activation include two pairs, Y348/Y352 and Y525/Y526, within the interdomain-B and kinase domains, respectively. Syk activation is initiated when these Y-sites are phosphorylated by SFKs or when dually Y-phosphorylated, ITAM-containing membrane proteins recruit Syk via its two SH2-domains, followed by Syk autophosphorylation.

Whereas other major serine/threonine or tyrosine protein kinases such as PKC or SFKs have multiple family members expressed in human platelets, Syk has a crucial position, since it is the only member of its family expressed in human and murine platelets [16,17]. The essential role of Syk is also supported by genetic evidence and its multifaceted regulation.

Mice embryos presenting with a homozygous targeted mutation in the *Syk* gene (by deletion of one exon of the *Syk* gene encoding for 41 residues in the Syk kinase domain in embryonic stem cells) die from severe hemorrhage before birth [27]. Mice lacking platelet Syk were protected from arterial thrombosis and ischemic stroke [28], highlighting the important role of Syk in platelets.

While the primary activation of Syk is tightly controlled by a few tyrosine-phosphorylation sites, there is increasing evidence, obtained mostly with murine and human B-cells, that Syk contains multiple additional tyrosine (Y), serine (S), and threonine (T) phosphorylation sites, which are thought to be important for recruiting additional regulatory proteins [19,20,29]. Phosphorylation of the interdomain-B site S297 (S291 in the murine protein) is documented in multiple phosphoproteomic databases, including human platelets [30,31], but further information is limited. When murine or human Syk was introduced into a chicken B cell model system (DT40), murine Syk S291 phosphorylation enhanced Syk coupling to the B-cell antigen receptor (BCR) [32], whereas human Syk S297 phosphorylation diminished antigen–receptor signaling [29].

Recently, we showed that specific activation of human platelets via GPVI (convulxin) or GPIbα (echicetin beads) not only stimulated transient Syk tyrosine phosphorylation and Syk activation, but also stoichiometric, transient, PKC-mediated phosphorylation of Syk S297 [33]. Moreover, pharmacological or protein kinase A (PKA)-induced PKC inhibition abolished this Syk S297 phosphorylation, but enhanced GPVI-/GPIbα-stimulated Syk tyrosine phosphorylation/activity, suggesting that Syk S297 phosphorylation is a mechanism for Syk feedback inhibition in human platelets. Importantly, the transient nature of Syk S297 phosphorylation clearly indicated the presence of a very active Syk pS297 protein phosphatase, which was not previously studied. Separately, we demonstrated the presence of a wide spectrum of serine/threonine protein phosphatases in human and murine platelets, including, surprisingly, the entire cell cycle check-point system “MAST(L)-ENSA/ARPP19-PP2A” [34]. Our observation of the transient nature of Syk S297 phosphorylation indicated the presence of an unknown Syk pS297 protein phosphatase. Therefore, we aimed to identify and elucidate the role of the serine/threonine protein phosphatase PP2A in regulating the tyrosine protein kinase Syk activity/tyrosine phosphorylation in washed human platelets. Experiments with specific pharmacological inhibition of PP2A showed that PP2A was significantly involved in Syk pS297 dephosphorylation in intact human platelets. PP2A inhibition alone led to stoichiometric Syk S297 phosphorylation without affecting Syk tyrosine phosphorylation. However, PP2A inhibition prolonged subsequent GPVI-mediated Syk S297 phosphorylation and strongly reduced Syk tyrosine phosphorylation and activity, indicating a novel regulatory mechanism of Syk by PP2A.

## 2. Results

### 2.1. PP2A Inhibition, but Not PP1 Inhibition, Results in Stoichiometric Syk S297 Phosphorylation in Platelets

**Figure 1:**
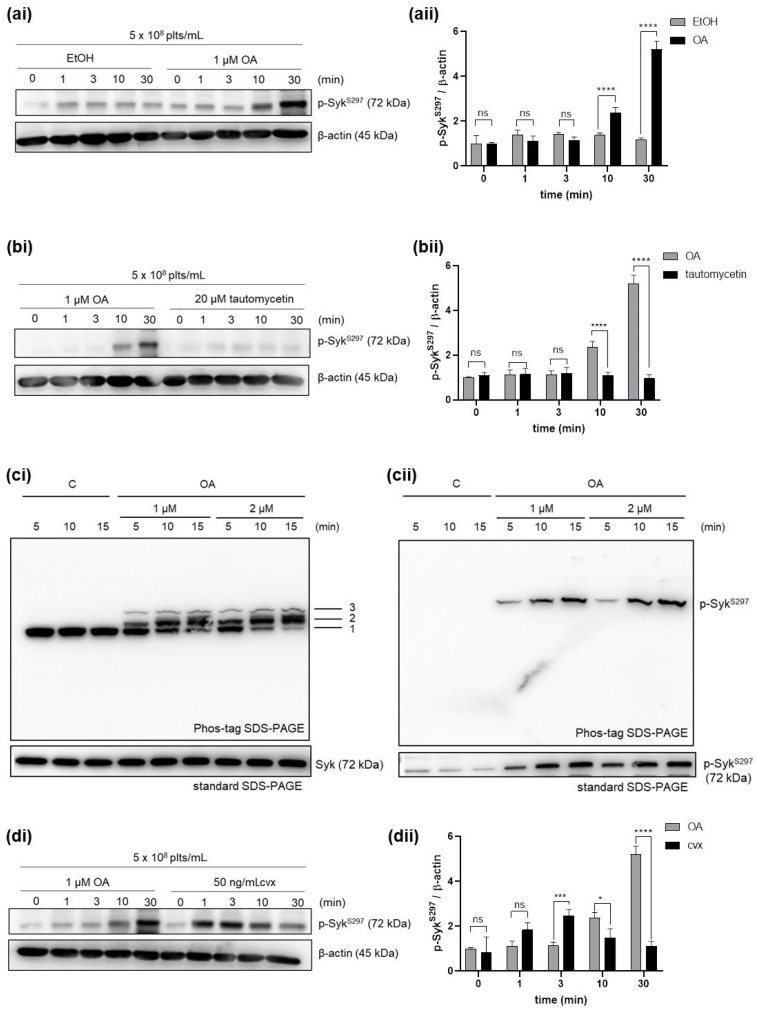
Specific protein phosphatase 2A (PP2A) inhibition elevates Syk S297 phosphorylation in human platelets. Washed human platelets were incubated, as indicated, (**a**,**c**) with the vehicle ethanol (EtOH; C: vehicle control), (**a**–**d**) with 1 µM/2 µM of the PP2A inhibitor okadaic acid (OA), or with (**b**) 20 µM of the PP1 inhibitor tautomycetin, or were stimulated with (**d**) 50 ng/mL convulxin (Cvx) at 37 °C. Samples were analyzed by (**a**–**d**) conventional SDS-PAGE (8.0% *w*/*v* polyacrylamide), or by (**ci**,**cii**) phos-tag SDS-PAGE (6.0% *w*/*v* polyacrylamide and 35 µM Zn^2+^-phos-tag). Time-dependent phosphorylation of Syk S297 was monitored and analyzed by immunoblotting compared to β-actin. (**ai**, **bi**, **di**) Representative western blots of Syk S297 in the presence of (**aii**) OA, (**bii**) tautomycetin, or (**dii**) Cvx. Quantitative data of Syk S297 in the presence of (**aii**) OA, (**bii**) tautomycetin, or (**dii**) Cvx are represented as means ± S.D from three technical experiments, with platelets from the same donor. * *p* < 0.1, *** *p* < 0.001, **** *p* < 0.0001, ns *p* > 0.05.

In previous studies, we observed that activation of both GPIbα or GPVI caused a strong but transient stimulation of Syk S297 phosphorylation, suggesting activity of both a very active Syk S297 protein kinase but also protein phosphatase [33]. In order to characterize this protein phosphatase further, we studied the effect of PP2A and protein phosphatase 1 (PP1) on the regulation of Syk phosphorylation using conditions recently established in our laboratory for human platelets [34]. When washed human platelets were pre-incubated with 1 µM of okadaic acid (OA), (PP2A inhibitor), time-dependent phosphorylation of Syk on S297 (Figure 1a) compared to vehicle control was observed. In contrast, pre-incubation with 20 µM of tautomycetin (PP1 inhibitor) had no effect on Syk S297 phosphorylation (Figure 1b). Previously, we showed that OA (0–2—1 µM) increased the phosphorylation of established PP2A substrates such as vasodilator-stimulated phosphoprotein (VASP) and the protein kinases Akt, p38, and ERK1/2 under these conditions [34]. Then, we investigated the quantitative effects of OA on Syk S297 phosphorylation using phos-tag SDS-PAGE analysis in combination with regular SDS-PAGE (Figure 1c). The phos-tag gel showed a near complete shift of a basal Syk band (#1) to a higher band (#2), with a corresponding loss of band #1 after 15 min of incubation with OA (1 and 2 µM). Therefore, PP2A inhibition caused stoichiometric and stable Syk phosphorylation (Figure 1(ci)), which closely correlated with Syk S297 phosphorylation, as analyzed by immunoblots of both phos-tag gels and regular SDS-PAGE gels (Figure 1(cii)).

We recently described time-dependent convulxin (Cvx), GPVI-mediated effects on Syk-activation and S297 phosphorylation [33]. Therefore, we compared the profile of Syk S297 phosphorylation induced by OA and Cvx, which differed significantly (Figure 1d). OA induced slow-onset, time-dependent Syk S297 phosphorylation which reached a maximum after 30 min. Previously, we observed similar time- and concentration-dependent effects of OA on several established PP2A substrates [34]. In contrast to OA, and as observed before, Cvx induced a transient (within one minute), GPVI-mediated Syk S297 phosphorylation with a rapid onset (Figure 1d).

### 2.2. Syk S297 Phosphorylation Induced by PP2A Inhibition Is Only Minimally Impaired by Syk or PKC Inhibitors

**Figure 2:**
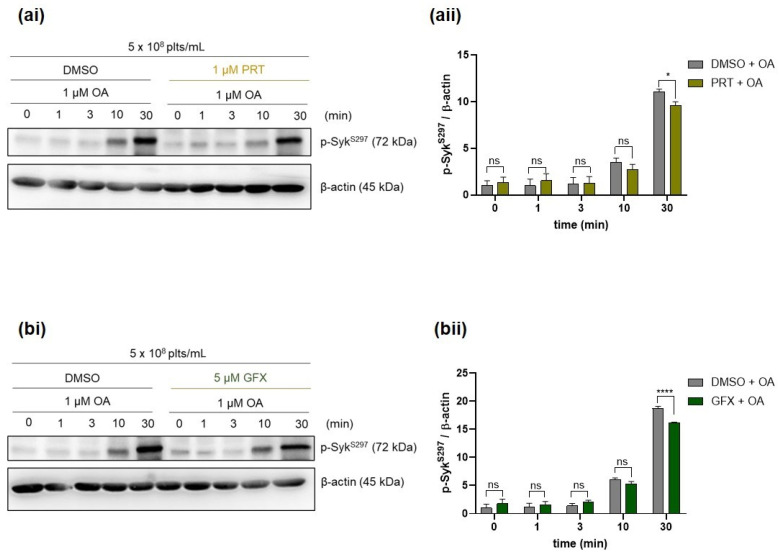
Inhibition of Syk or protein kinase C (PKC) has only minimal effects on OA-induced Syk S297 phosphorylation. Washed human platelets were preincubated, as indicated, for 5 min with (**a**,**b**) vehicle control (dimethyl sulfoxide, DMSO), with (**a**) 1 µM of a Syk inhibitor (PRT; PRT318), or with (**b**) 5 µM of a PKC inhibitor (GFX; 2-[1-(3-dimethylaminopropyl)indol-3-yl]-3-(indol-3-yl) maleimide) prior to the addition of 1 µM OA at 37 °C. Samples for western blot analysis were taken at the indicated time points after the addition of OA and mixed with Laemmli buffer. Time-dependent phosphorylation of Syk S297 was monitored and analyzed by immunoblotting compared to β-actin. (**ai**, **bi**) Representative western blots. of Syk pS297 in the presence of (**ai**) the Syk inhibitor and (**bi**) the PKC inhibitor Quantitative data of Syk pS297 in the presence of (**aii**) the Syk inhibitor and (**bii**) the PKC inhibitor are represented as means ± S.D from three technical experiments, with platelets from the same donor. * *p* < 0.1, **** *p* < 0.0001, ns *p* > 0.05.

Previously, we showed by conventional SDS-PAGE plus phos-tag analysis that the transient, Cvx-induced phosphorylation of Syk S297 was stoichiometric and mediated by both Syk itself and PKC [34]. To investigate the possible roles of both Syk and PKC for the effect of PP2A inhibition on Syk S297 phosphorylation, we used two well-established inhibitors targeting Syk (PRT) and PKC (GFX). In contrast to our previous results with Cvx-stimulation [33], OA-induced Syk S297 phosphorylation was only minimally affected by the Syk inhibitor (Figure 2a) and by the PKC inhibitor (Figure 2b), suggesting that a protein kinase other than PKC mediates the OA-induced Syk S297 phosphorylation.

### 2.3. PP2A Inhibition Significantly Reduces Cvx-Stimulated Syk Y525/526 Phosphorylation (Kinase Domain)/Activity but Does Not Inhibit Cvx-Stimulated Syk S297 Phosphorylation (Interdomain-B)

**Figure 3:**
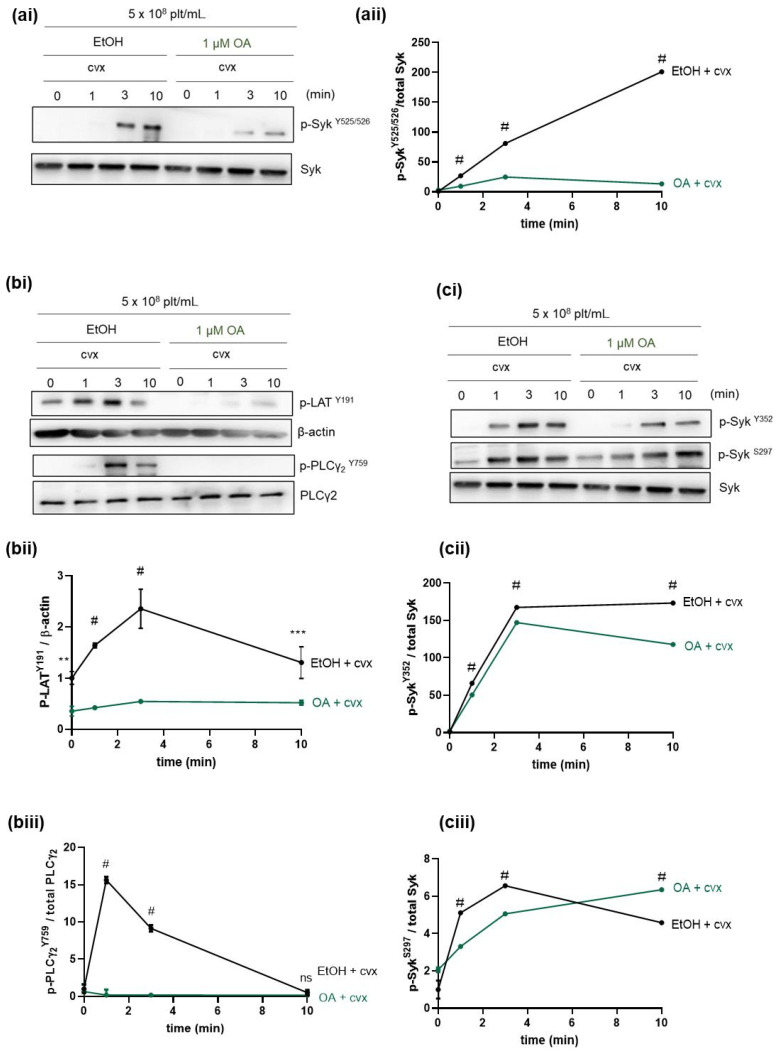
PP2A inhibition significantly reduced Cvx-stimulated Syk Y525/526 phosphorylation (kinase domain) and phosphorylation of Syk substrates LAT and PLCγ2, with minimal effects on the phosphosites Syk Y352 and S297 (both interdomain-B). Washed human platelets were pre-incubated for 10 min with vehicle control (EtOH) or with 1 µM of the PP2A inhibitor OA prior to stimulation with 50 ng/mL Cvx. Samples for western blot analysis were taken at the indicated time points after the addition of Cvx and mixed with Laemmli buffer. Time-dependent phosphorylation of (**a**) Syk Y525/526 (**b**), LAT Y191 and PLCγ2, and (**c**) Syk Y352 and S297 was analyzed by immunoblotting compared to the corresponding loading control. (**ai**, **bi**, **ci**) Representative western blots of Syk Y525/526 (**ai**), LAT Y191, PLCγ2 Y759 (**bi**), Syk Y352, Syk S297 (**ci**). Quantitative data of (**aii**) Syk Y525/526, (**bii**) LAT Y191, (**biii**) PLCγ2 Y759, (**cii**) Syk Y352, and (**ciii**) Syk S297 are represented as means ± S.D from three technical experiments, with platelets from the same donor. ** *p* < 0.01, *** *p* < 0.001, ^#^ *p* < 0.0001, ns *p* > 0.05.

We aimed to study the effect of PP2A inhibition on Cvx-induced Syk phosphorylation and Syk activity (Figure 3). PP2A inhibition significantly reduced Cvx-mediated Syk Y525/526 phosphorylation, a phosphosite within the kinase domain and marker of Syk activity (Figure 3a). In order to extend this finding, we analyzed Cvx-induced phosphorylation of the established Syk substrates, linker of activated T cells (LAT) and PLCγ2 on Y191 and Y759, respectively (Figure 3b). The phosphorylation of these two substrates was essentially abolished by the OA pretreatment.

**Figure 4:**
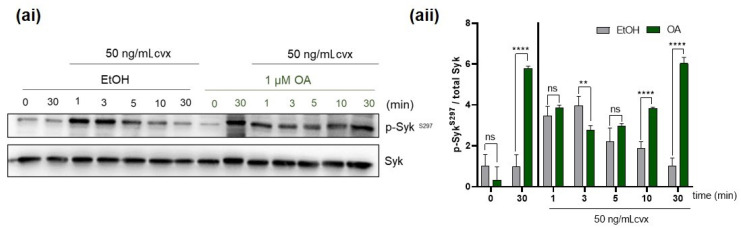
PP2A inhibition significantly increased Cvx-mediated Syk S297 phosphorylation. Washed human platelets incubated with vehicle control (EtOH) or with 1 µM of the PP2A inhibitor OA before 50 ng/mL Cvx were added. Samples for western blot analysis were taken after the indicated time points after the addition of Cvx and mixed with Laemmli buffer. Time-dependent phosphorylation of Syk S297 was monitored and analyzed by immunoblotting compared to total Syk. (**ai**) Representative western blot of Syk S297 induced by Cvx in the presence or abcence of 1 µM OA. Quantitative data (**aii**) of Syk S297 are represented as means ± S.D from four different experiments (two biological and two technical replicates), with platelets from three different donors. ** *p* < 0.01, **** *p* < 0.0001, ns *p* > 0.05.

Interestingly, the inhibition of PP2A under these conditions showed only minimally inhibitory effects on the Cvx-stimulated phosphorylation of Syk Y352 and S297, which are both located in the interdomain-B of Syk (Figure 3c). However, these data (Figure 3ci,cii and Figure 4) showed that OA pretreatment prolonged Cvx-induced Syk S297 phosphorylation.

### 2.4. Extended PP2A Inhibition Prevents Down-Regulation of and Prolongs Cvx-Stimulated Syk S297 Phosphorylation

We aimed to extend our observations on the direct effect of OA on Syk S297 phosphorylation evoked by Cvx. Therefore, we pretreated washed platelets with vehicle control (EtOH) or with OA (1 µM) for 30 min, and then with Cvx (50 ng/mL). We previously showed that Cvx induces transient S297 phosphorylation. Our data here demonstrate that PP2A inhibition prolongs Syk S297 phosphorylation up to 30 min after Cvx stimulation (Figure 4), best seen at the time points 5–30 min after Cvx addition. The trough-like appearance of Syk S297 phosphorylation in the OA/Cvx combination (Figure 4aii) suggested that two components of S297 phosphorylation were present, one stimulated by the Cvx/Syk-kinase dependent PKC activation [33] and one by the alternative kinase system activated by OA. However, the OA pretreatment inhibited Syk activity (Figure 3, also Supplementary Figure S1), and may therefore partially reduce Syk S297 phosphorylation in the early phase after Cvx stimulation.

Samples from the same experiment shown in Figure 4 were also analyzed for Y-phosphorylation of Syk and Syk substrates (Supplementary Material Figure S1). Incubation of platelets with OA prior Cvx stimulation strongly inhibited Syk Y525/526 (Supplementary Figure S1a) and Syk Y352 phosphorylation (Supplementary Material Figure S1b). In order to validate the functional outcome of this inhibitory effect, we also studied the Y-phosphorylation of the Syk substrates LAT and PLCγ2 (Supplementary Material Figure S1c). Phosphorylation of LAT Y191 and of PLCγ2 Y759 was strongly inhibited (Supplementary Material Figure S1ci–ciii).

These results show that PP2A inhibition in human platelets differentially regulates Syk serine and tyrosine phosphorylation and reduces Cvx-stimulated Syk activity in human platelets.

## 3. Discussion

The Syk tyrosine protein kinase is essential for the activation and subsequent responses of platelets and immune cells. Syk is also known to be tightly regulated by multiple tyrosine and also serine/threonine protein kinases with their multiple phosphorylation sites. Very recently, we demonstrated and reported for the first time that GPVI-induced activation of human platelets caused a rapid, stoichiometric, transient phosphorylation of Syk S297, which is located at the crucial Syk interdomain-B [33]. These and additional data led us to propose that Syk S297 phosphorylation represents a so-far unknown mechanism of feedback inhibition. Since important phosphorylation-based regulatory mechanisms are controlled by both protein kinases and the corresponding protein phosphatases; since nothing is known about a Syk pS297 protein phosphatase in platelets and other cells, it was the major objective of this study to identify this protein phosphatase and its possible functional effects on Syk in human platelets.

Our present data show that incubation of intact human platelets with 1 µM okadaic acid (OA) caused the time-dependent appearance of Syk pS297, as detected by phosphosite-specific antibodies combined with regular western blot and phos-tag analyses (Figure 1a–c). Several lines of evidence suggested that this OA effect was due to the specific inhibition of PP2A. With purified enzymes or cell lysates, including lysates from human platelets, OA is an extremely potent inhibitor of the heterotrimeric PP2A (IC_50_~0.2 nM), directly binding to and inhibiting the PP2A catalytic subunit C, but other protein phosphatases only at much higher concentrations [34,35,36]. In studies with intact cells, such as platelets, membrane permeability properties of OA [37] and the intracellular concentration of PP2A catalytic subunits and other protein phosphatases are key factors which determine the potency and specificity of OA [34]. Human platelets contain the PP2A catalytic subunits Cα and Cβ (combined intracellular concentration of ~1 µM), one regulatory (scaffolding) subunit A (~1.7 µM) and several B regulatory subunits (B55α, B55δ, B56α, B56β, B56γ, B56δ and B56ε; each between 0.2 and 0.4 µM), which may form up to 14 different heterotrimeric holoenymes [34]. In agreement with these data and the PP2A properties, OA concentrations between 0.2 and 1 µM and incubation times between 10 and 30 min effectively inhibited PP2A activity, as indicated by the increased phosphorylation of established PP2A targets, such as vasodilator-stimulated phosphoprotein (VASP), RAC-alpha serine-threonine-protein kinase (Akt), mitogen-activated protein kinase p38 (p38), extracellular signal regulated kinase1 (ERK1), and α-endosulfine (ENSA). As a functional control, we showed that OA concentrations up to 1 µM did not inhibit α-thrombin-induced platelet shape change, whereas this was abolished by the protein phosphatase 1 (PP1) inhibitor tautomycetin. Platelet shape change, mediated by myosin light chain phosphorylation, requires the concerted regulation of myosin light chain kinase and its opponent, myosin light chain phosphatase, a specialized PP1 [35]. In the present study, the PP1 inhibitor tautomycetin (20 µM) did not affect Syk S297 phosphorylation. We conclude that OA-induced Syk S297 phosphorylation is due to PP2A inhibition, and that PP1 inhibition by tautomycetin does not affect this phosphorylation site.

The combined analysis of Syk S297 phosphorylation by regular western blots and by phos-tag analysis (Figure 1c) showed that a 10–15 min incubation of human platelets with OA strongly (1 µM) or almost completely (2 µM) shifted Syk from a lower band (#1; representing the Syk S297) to a higher band (#2; representing Syk pS297). With other conditions but related analyses (phosphoantibodies, phos-tag analysis), we recently showed that both GPVI stimulation (by convulxin) and GPIbα-stimulation (by echicetin beads) caused a rapid, stoichiometric, transient Syk S297 phosphorylation. The increase in Syk S297 phosphorylation was completely prevented by prior PKA activation, by blocking the secondary mediators thromboxane A2 and ADP, and by selective Syk inhibitors (PRT) or PKC inhibitors (GFX), but was strongly stimulated by the potent global PKC activator (PDBu). We concluded that one of the conventional PKCs was responsible for the GPVI/GPIbα-induced Syk S297 phosphorylation [33]. Our present data show a delayed but persistent OA response on Syk S297 phosphorylation, which clearly differs from the rapid but transient Cvx-induced effects, although stoichiometric changes (near complete shift in phos-tag gels) were achieved by both conditions. The quantitative aspects of the Syk S297 phosphorylation observed here were supported by previous phosphoproteomic studies reporting this site as one of the strongest, detectable phosphosites of Syk, but also showing additional sites [29,30]. In addition to the major OA-shifted-band in phos-tag gels (#2, Figure 1(ci)), a minor OA-shifted Syk-band (#3) was also noticed, which may represent a second, low-stoichiometric serine/threonine Syk phosphosite affected by PP2A inhibition.

The phosphorylation state of a given protein phosphosite, here, Syk S297, in intact cells is determined by the equilibrium of the corresponding protein kinase and protein phosphatase activity using this site as the substrate. Although there is some basal protein phosphorylation in resting platelets as detected by phosphoproteomics [30], most regulated protein phosphosites are present as dephosphoforms under these conditions, indicating that the protein phosphatase capacity exceeds that of protein kinases [31,34]. One can shift the phosphorylation state of Syk S297 toward phosphorylation by either activation of responsible protein kinases, as previously done, or by inhibition of the responsible protein phosphatase(s), as now reported. Here, we also addressed the question regarding which protein kinases phosphorylate Syk S297 when PP2A is inhibited. Surprisingly, inhibitors of Syk itself (PRT) or of PKC (GFX), which abolished echicetin beads/GPIbα- and convulxin/GPVI-stimulated Syk S297 phosphorylation [33], showed very little effect on OA- induced Syk S297 phosphorylation. These data indicate that there are at least two different Syk S297 protein kinases in platelets, i.e., a conventional PKC activated in response to several distinct receptor-dependent pathways (ADP, GPIbα, and GPVI), and a second protein kinase system whereby PP2A is inhibited. We reported that PP2A inhibition by OA, under similar conditions, resulted in phosphorylation and activation of several distinct protein kinases including Akt, MAST(L), p38, and ERK 1/2 [34]. From this list, Akt is a very interesting protein kinase candidate to promote Syk S297 phosphorylation, since it is also activated during GPIbα-mediated platelet activation by a phosphoinositide-3-kinase (PI3K)-dependent mechanism [38]. Furthermore, studies with B-cells suggested that Akt and 14-3-3 adapter proteins downregulate the activity of several B-cell receptor (BCR)-associated components, including bruton tyrosine kinase (BTK), B-cell linker protein (BLNK), and Syk, and inhibit Syk’s interaction with importin 7 [39,40].

The effects of PP2A inhibition on Syk S297 phosphorylation were then compared with the effects on Syk tyrosine phosphorylation and Syk activity by studying the Syk pY352 and pY525/526 phosphosites (as markers of Syk activation) and the tyrosine phosphorylation of the Syk substrates LAT (pY191) and PLCγ2 (pY759) as markers of Syk activity. We previously showed that PKC inhibition prevented GPIbα-/GPVI-induced Syk S297 phosphorylation but, in parallel, enhanced Syk Y352 and Y525/526, as well as LAT Y191 and PLCγ2 Y759 phosphorylation. This PKC-induced Syk S297 phosphorylation was proposed to be a mechanism of feedback inhibition [33]. Our present data, obtained using a completely different signaling approach, supported this conclusion. Short-term (10 min) and long-term (30 min) incubation with OA (1 µM) alone did not increase any of the very low (basal) levels of the tyrosine phosphorylation studied (Syk Y352, Y525/526; LAT Y191, PLCYγ2 Y759). In contrast, 10 min and 30 min pre-incubations with OA (1 µM) strongly inhibited and almost abolished the effects of Cvx on these Syk and LAT/PLCγ2 tyrosine phosphorylation sites. However, OA strongly increased Syk S297 phosphorylation, which remained elevated throughout the entire incubation time, up to 60 min including the co-incubation with Cvx. Alone, Cvx produced a strong but transient Syk S297 phosphorylation.

Our results showed that PP2A inhibition differentially regulates Syk serine (↑ pS297) and tyrosine phosphorylation sites (↓ pY352, pY525/526) and that PP2A inhibition reduces Cvx-stimulated Syk activity in human platelets. These effects are a mirror-image, at least in part, of our studies with pharmacological and physiological PKC inhibitors during Cvx-induced platelet activation, which were shown to abolish Syk S297 phosphorylation but enhance Syk tyrosine phosphorylation and activity [33]. The pronounced, reversible regulation of Syk S297 phosphorylation by several distinct serine/threonine protein kinases and phosphatases with significant effects on Syk activity strongly suggests that this phosphosite and/or its location is a crucial but indirect regulator of this tyrosine kinase. From the many Syk tyrosine phosphorylation sites known, the two pairs of Y348/Y352 (interdomain-B) and Y525/526 (kinase domain) are closely associated with Syk activation/kinase activity and are predominantly phosphorylated by SFKs or Syk autophosphorylation, respectively. Mechanistically, mutation of the Y525/526 sites strongly inhibited but did not abolish kinase activity, whereas mutation of Y348/Y352 of the interdomain-B did not affect kinase activity but did eliminate binding of the Syk substrate PLCγ2, which also eliminated tyrosine phosphorylation of this protein by Syk [22,23,41]. Interestingly, GPVI-induced Syk activation was suppressed by the protein tyrosine phosphatase-TULA-2 (TULA-2) protein tyrosine phosphatase, which selectively dephosphorylated Syk Y346 (Y352 in human Syk) in murine platelets [42]. Multiple mutagenesis studies supported the concept of autoinhibitory interactions of the Syk kinase domain and interdomain-B, which can be disrupted either by binding of tyrosine-phosphorylated ITAM proteins to the Syk SH2 domains and/or by tyrosine phosphorylation of sites within the interdomain-B (Y348/Y352), resulting in Syk autophosphorylation at Y525/526 and activation [20,22]. This hypothesis is strongly supported by the crystal structures of full-length Syk (fl-Syk) as wild-type and Y348F/Y352F (YYFF) mutant forms in complex with the nonhydrolyzable ATP-analogue AMP–PNP, demonstrating the molecular nature of the autoinhibited conformation [24]. These impressive structures of fl-Syk, combined with further kinetic data [43], clearly demonstrated the existence of an autoinhibited Syk, but also suggested crucial role(s) of interdomain-B, including Y348/Y352 for the activation of Syk. Unfortunately, a considerable part (amino acids 262–337, containing other phosphorylation sites including S297) of the crucial interdomain-B was not resolved, perhaps due to the particularly flexible nature of this component [24]. It is tempting to speculate that Syk S297 phosphorylation stabilizes the autoinhibited conformation of Syk, whereas Syk Y348/Y352 phosphorylation stabilizes the active kinase form. Furthermore, additional Syk phosphorylation sites, as well as other covalent modifications and adapter proteins, may participate in this regulation [29,44]. The role of human and murine Syk interdomain-B phosphorylation, including serine 297 (human)/S291 (murine) Syk was previously investigated by two groups using the chicken B-cell model system DT40, but the functional effects on BCR signaling differed between murine and human Syk [29,32]. These differences are not yet clarified and are perhaps due to different adapter proteins recruited, namely, the chaperone prohibitin by murine Syk and the adapter protein 14-3-3γ by human Syk, which may also affect Syk recruitment to ITAM proteins of plasma membranes.

Clearly, there are limitations of these present studies. We did not identify the precise mechanism by which Syk S297 phosphorylation/dephosphorylation controls Syk activity. Future studies should analyze the precise molecular role of the Syk S297 phosphosite using structural, mutational, and further cellular/functional approaches, since it represents a regulatory hotspot for a tyrosine kinase crucial for the function of platelets and many immune cells. Since Syk is controlled by multiple post-translational modifications and protein interactions [29,44], it is possible that such regulatory mechanisms differ between species and cell types.

**Figure 5:**
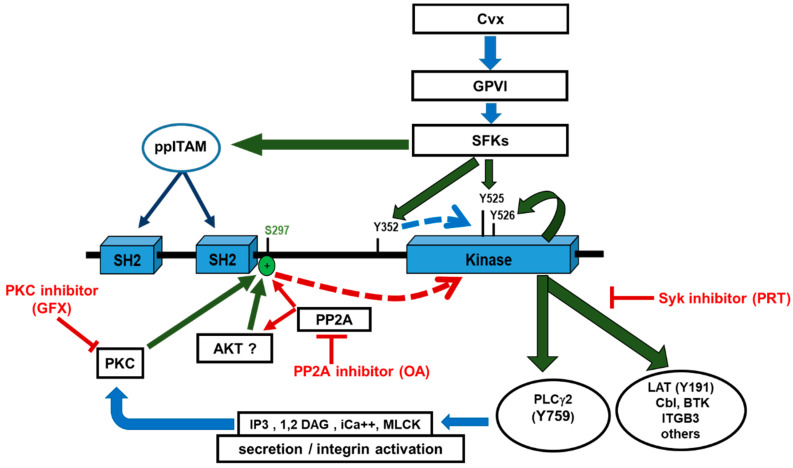
Model showing the regulation of Syk by the serine/threonine protein phosphatase PP2A in human platelets. GPVI ligands such as convulxin (Cvx) activate platelets by src family kinase (SFK)-induced Syk stimulation. SFKs cause dual phosphorylation of proteins with “immunoreceptor tyrosine-based activation motifs” (ppITAMs), which then recruit Syk via its two src homology 2 (SH2) domains to the platelet cell membrane. This alters the Syk conformation, resulting in Syk activation and phosphorylation at Y352 (interdomain-B) and at Y525/526 (kinase domain), predominantly by SFKs and autophosphorylation, respectively. Fully activated Syk catalyzes Y-phosphorylation of specific substrates and regulatory proteins, such as phospholipase Cγ2 (PLCγ2), linker for activation of T-cells (LAT), E3 ubiquitin-protein ligase CBL (Cbl), bruton tyrosine kinase (BTK), and integrin beta-3 (ITGB3), often organized as “signalosome” complexes. Y-phosphorylation-induced activation of PLCγ2 increases the release of ADP (from platelet δ-granules) and TxA2 via elevation of InsP3 and Ca^2+^. GPVI activation not only induces Syk Y-phosphorylation, but also PKC-mediated Syk phosphorylation at S297, within the interdomain-B in the vicinity of the SH2 domain. Inhibition of PP2A by okadaic acid (OA) leads to stoichiometric Syk S297 phosphorylation and prevents Syk pS297 dephosphorylation after GPVI stimulation, indicating that PP2A is the major protein phosphatase responsible for Syk pS297 dephosphorylation. Syk S297 phosphorylation negatively correlates with Syk Y-phosphorylation (Y352, Y526/Y526) and Syk substrate (LAT, PLCγ2) phosphorylation. In human platelets, the Syk S297 site shuttles between a completely phosphorylated form, catalyzed by PKC and potentially by Akt, and a completely dephosphorylated form, achieved by PP2A. It is suggested that this switch modulates Syk kinase Y-phosphorylation and activity in platelets. Induced phosphorylation, green arrows; inhibition, red arrows; indirect effect, dashed line; interaction/stimulation, blue arrows.

A remarkable result of our studies with human platelets is that Syk S297 phosphorylation shuttles between complete PP2A-catalyzed dephosphorylation and complete phosphorylation, induced by pathways activating PKC, Akt, and perhaps additional protein kinases (see Figure 5 as a model).

Another limitation of these present studies is that we identified PP2A as Syk pS297 protein phosphatase, but did not identify the PP2A subtype(s) responsible for this activity. PP2A is not a single enzyme but a large protein family, with more than 92 different heterotrimeric forms present in humans [45,46] and 14 heterotrimeric forms expressed at the protein level in human platelets [34]. Recent advances established that distinct properties of the individual PP2A heterotrimers, such as substrate specificity, cellular localization, and regulation by endogenous inhibitors, are primarily determined by the PP2A regulatory subunits [47,48]. Importantly, PP2A activity is often impaired in cancer, inflammation, and other diseases [46,49]. Due to the enormous heterogeneity and possible redundancy of the PP2A heterotrimers, it was very difficult in the past to address and elucidate PP2A functions at the molecular level. However, recent progress in developing reagents which specifically inhibit or stimulate individual heterotrimers [50,51] appears to be paving the way for such studies in the future.

This study established an important interaction between the PP2A family and the tyrosine kinase Syk in human platelets, which is also likely to be significant regarding the regulation of immune cells and other tyrosine kinases, thereby requiring further investigation. This S297 site regulated by phosphorylation/dephosphorylation is located within the N-terminal Syk interdomain-B, whereas the well-established Y348/Y352 phosphosites important for activation are located within the C-terminal part of this linker domain. Overall, this Syk interdomain-B is an important molecular switch, which could allow graduated levels of Syk activity, depending on the state of interdomain and kinase domain phosphorylation. Such graduated activity of Syk was suggested to be important for tonic signaling, where complete or long-term Syk activation may not be desirable [43]. Syk was established as a promising therapeutic target in autoimmunity and inflammation [52]. A full elucidation of the molecular regulation of the Syk interdomain-B and its functional effects may be helpful to understand the cellular effects of current and future Syk inhibitors.

## 4. Materials and Methods

### 4.1. Blood Donors and Ethics Approval

Blood was collected from 10 sex-matched, healthy volunteers (age 24–70 years) without taking platelet-affecting drugs and without presenting inflammatory, infectious, or allergic symptoms for at least 14 days, respectively, including normal blood counts. The health statuses of volunteers were routinely approved by the local medical office of the University Medical Center Main. All blood donors gave their informed consent. This study was approved in accordance with the Declaration of Helsinki by the local Ethics Committee of the University Medical Center Mainz (Study No. 837.302.12;25.07.12; 2018-13290_1;27.07.2018).

### 4.2. Preparation and Stimulation of Human Platelets

Human platelets were washed and isolated as previously described [33,34,38,53]. Briefly, citrated blood from healthy volunteers supplemented with 2 mM ethyleneglycol- *bis*(β-aminoethyl)-N,N,N′,N′-tetraacetic acid (EGTA) was centrifuged at 200× *g* for 10 min at room temperature (RT) to generate platelet-rich plasma (PRP). PRP was diluted 1:1 with citrate-glucose-sodium (CGS) washing buffer (120 mM NaCl, 12.9 mM trisodium citrate dihydrate, 30 mM D-glucose, pH 6.5) and centrifuged at 69× *g* for 10 min at room temperature (RT) to pellet the leukocytes. The supernatant was centrifuged at 400× *g* for 10 min at RT to pellet the platelets. Each platelet pellet was resuspended in 3 mL CGS buffer and centrifuged at 400× *g* for 10 min at RT. Finally, platelets were resuspended in 4-(2-hydroxyethyl)-1-piperazineethanesulfonic acid (HEPES) buffer (150 mM NaCl, 5 mM KCl, 1 mM MgCl_2_·8 H_2_O, 10 mM D-glucose, 10 mM HEPES, pH 7.4). The platelet count was adjusted to 5 × 10^8^/mL and platelets were kept at 37 °C prior to treatment, which was also performed at 37 °C without stirring. A higher platelet count was used because the OA preincubation was already established with higher platelet counts and worked well for these conditions. For comparable results, all experiments needed to be done with the platelet count of 5 × 10^8^/mL.

### 4.3. SDS-PAGE, Zn^2+^ Phos-tag^tm^ -SDS-PAGE and Western Blot Analysis

Washed platelets were prepared in one stock and separated for treatment. They were treated at 37 °C without stirring. Washed platelets were pre-incubated with 1 µM OA or EtOH (vehicle control), 20 µM tautomycetin for 10 min, or with 1 µM PRT or 5 µM GFX or DMSO (verhicle control) for 5 min at 37 °C. Pretreated samples were stimulated/inhibited with 50 ng/mL convulxin or 1 µM/2 µM OA or EtOH. Samples were taken after the corresponding time points and added to a 1 mL tube already containing 3× Laemmli buffer (200 mM tris/HCl, 15% (*v*/*v*) glycerol, 6% (*w*/*v*) SDS, 0.06% (*w*/*v*) bromphenol blue; 1:10 β-mercaptoethanol) in a ratio of 1:10. Samples were boiled at 95 °C for 10 min under gentle shaking. Platelet proteins were separated by electrophoresis using 8% SDS-polyacrylamide gels or Zn^2+^ Phos-tag^tm^-SDS-PAGE (6% (*v*/*v*) acrylamide, 35 µM Zn^2+^-phos-tag) followed by immunoblotting, as previously described [33,34]. Zn^2+^ Phos-tag^tm^-SDS-PAGE separated proteins according to their phosphorylation ratio, so only an antibody against the total protein was necessary. After transfer, polyvinylidene difluorid (PVDF)-membranes were blocked with 5% bovine serum albumin (BSA) in 20 mM tris, 140 mM NaCl, 0.1% (*v*/*v*) Tween^®^-20 (Bio-Rad Laboratories GmbH, Feldkirchen, Germany, 1xTBST), pH 7.4) for 1 h at RT and incubated overnight with the respective antibodies in 5% BSA 1xTBST at 4 °C. Membranes were washed three times with 1xTBST and incubated for 1 h at RT with the secondary-horseradish peroxidase (HRP)conjugated antibodies in 5% BSA–TBST. After three washes with 1xTBST, membranes were developed by electrochemiluminescence (ECL) detection. Phosphoantibodies against Syk S297, Y525/526, Y352, LAT Y191, PLCγ2 Y759, and β-actin (Cell Signaling Technologies, Danvers, MA, USA) or anti-Syk, anti-PLCγ2 (Santa Cruz Biotechnology, Santa Cruz, CA, USA) were used, as previously described [33].

### 4.4. Statistical Analysis

Each data set represents 3 different technical experiments performed with platelets from the same healthy volunteer, unless stated differently. Data are represented as means ± S.D. Stastistical analysis was performed using GraphPad Prism 8 (GraphPad Software, San Diego, CA, USA). Two-way ANOVA, followed by Sidak’s multiple comparison test for comparison of two groups at the same time point was used. *p* < 0.05 was considered as significant.

## Supplementary Materials

The following is available online at https://www.mdpi.com/1422-0067/21/23/8939/s1. Figure S1: PP2A inhibition significantly inhibited the transient phosphorylation of Syk Y525/526, Y352 stimulated by Cvx.

## Abbreviations

ADP	Adenosine diphosphate
BLNK	B-cell linker protein
BTK	Bruton tyrosine kinase
cAMP	Cyclic adenosine monophosphate
cGMP	Cyclic guanosine monophosphate
Clec-2	C-type lectin 2
Cvx	Convulxin
GFX	GF109203X; 2-[1-(3-Dimethylaminopropyl)indol-3-yl]-3-(indol-3-yl) maleimide
ITAM	Immunoreceptor tyrosine-based activation motif
LAT	Linker adaptor for T-cells
PDBu	Phorbol 12, 13-dibutyrate
PKC	Protein kinase C
PLCγ2	Phospholipase Cγ2
PRT	PRT060318; 2-(1R,2S)-2-aminocyclohexylamino)-4-(m-tolylamino)pyrimidine-5-carboxamide
S	Serine
Syk	Spleen tyrosine kinase
T	Threonine
TxA2	Thromboxane A2
vWF	Von Willebrand factor
Y	Tyrosine
C	Control
PP2A	Protein phosphatase 2 A
PP1	Protein phosphatase 1
OA	Okadaic acid
EtOH	Ethanol
