# Supplementary material for: The Serine/Threonine Protein Phosphatase 2A (PP2A) Regulates Syk Activity in Human Platelets
Source: Int J Mol Sci. 2020 Nov 25;21(23):8939. doi: 10.3390/ijms21238939 (PMC7728356; doi:10.3390/ijms21238939)
Supplement: Supplementary file 1 [file ijms-21-08939-s001.pdf]

International Journal of
Molecular Sciences

Supplementary Material

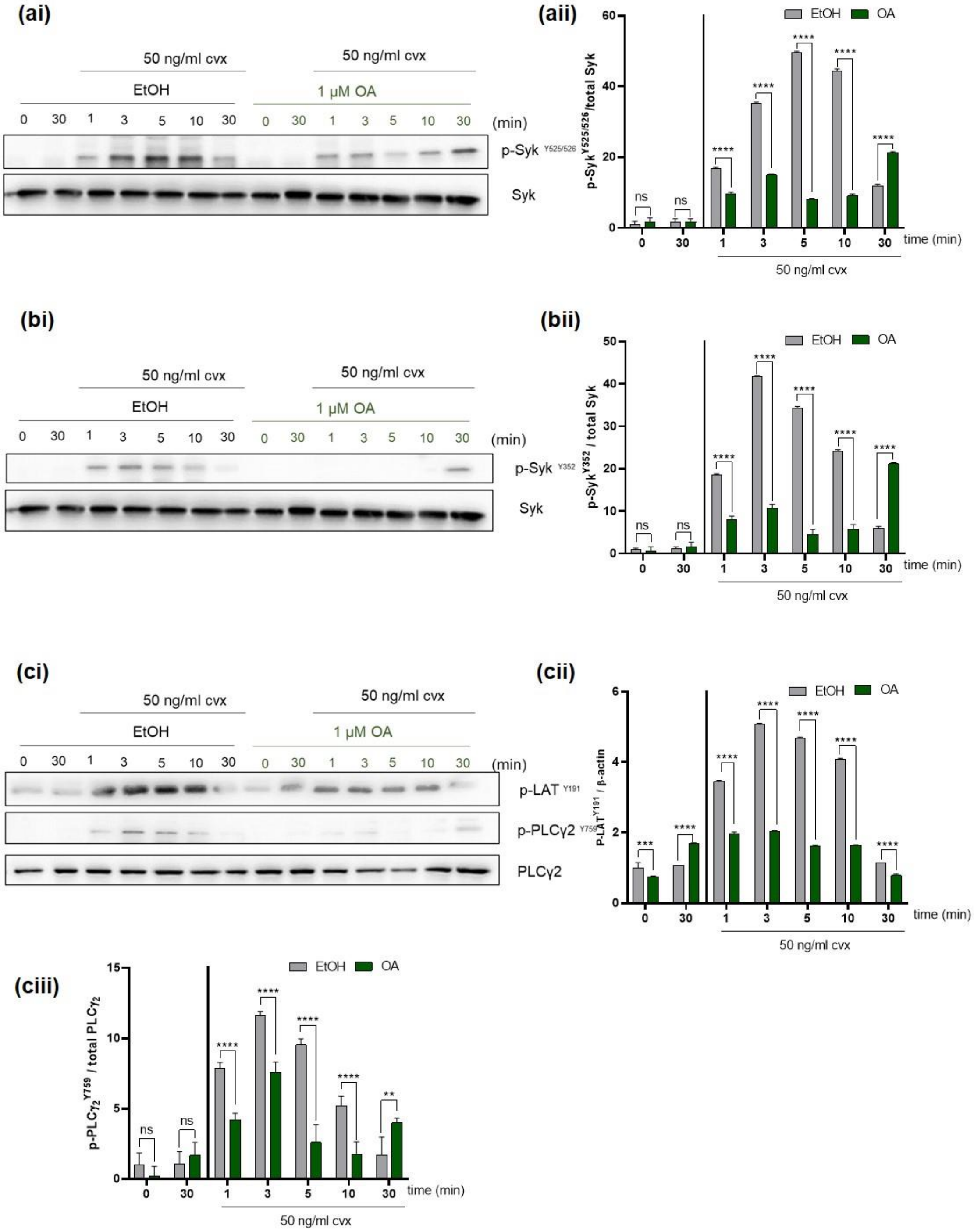

Figure S1. PP2A inhibition significantly inhibited the transient phosphorylation of Syk Y525/526, Y352 stimulated by Cvx. Washed human platelets were incubated with vehicle control (EtOH) or with 1μM of the PP2A inhibitor OA for 30 min, followed (in both cases) by the addition of 50ng/mL Cvx. Samples for western blot analysis were taken at the indicated time points after the addition of Cvx and mixed with Laemmli buffer. Time-dependent phosphorylation of (**ai**) Syk Y525/526, (**bi**) Syk Y352, (**ci**) LAT and PLCγ2 was analyzed by immunoblotting (representative western blots) compared to the corresponding loading control. Quantitative data (**aii**, **bii**, **cii**, **ciii**) are represented as means ± S.D from 4 different experiments (2 biological and 2 technical replicates) with platelets from three different donors. **p<0.01, ****p<0.0001, ns p>0.05.
